# Patient reported outcomes and return to play after arthroscopic hip labral reconstruction in athletes: a systematic review and meta-analysis

**DOI:** 10.1093/jhps/hnaf067

**Published:** 2025-11-24

**Authors:** Deniz C Ince, Kyle P O’Connor, Mia S Hagen, John C Clohisy, Jeffrey J Nepple

**Affiliations:** Department of Orthopaedic Surgery, Washington University School of Medicine, Washington University in St. Louis, Campus Box 8233, 660 S. Euclid Ave., Saint Louis, MO 63110, USA; Department of Orthopaedic Surgery, Washington University School of Medicine, Washington University in St. Louis, Campus Box 8233, 660 S. Euclid Ave., Saint Louis, MO 63110, USA; Department of Orthopaedic Surgery and Sports Medicine, University of Washington, 3800 Montlake Blvd NE, Campus Box 354060, Seattle, WA 98195, USA; Department of Orthopaedic Surgery, Washington University School of Medicine, Washington University in St. Louis, Campus Box 8233, 660 S. Euclid Ave., Saint Louis, MO 63110, USA; Department of Orthopaedic Surgery, Washington University School of Medicine, Washington University in St. Louis, Campus Box 8233, 660 S. Euclid Ave., Saint Louis, MO 63110, USA

## Abstract

Femoroacetabular impingement is a common cause of labral damage and hip pain in athletes. In severe labral tears with inadequate healthy labrum for repair, labral reconstruction has shown positive clinical outcomes in the general population. This study aimed to conduct a systematic review and meta-analysis on athletes undergoing arthroscopic hip labral reconstruction, focusing on postoperative patient-reported outcomes (PROs) and return to play (RTP). A systematic review of the literature was performed to include studies reporting PROs of labral reconstruction in athletes across multiple databases. Of the initial 430 identified records, four satisfied inclusion criteria of reporting PROs at 1-year minimum follow-up and RTP. Athletes included recreational through professional levels. Three studies analyzed return to same level of play (RTSP). Meta-analysis was conducted by pooling data, creating forest plots, and reporting odds ratios (OR). The four studies included 126 hips with average age at surgery of 32.2 years. All PROs in each study showed significant improvements with postoperative pooled weighted improvements in modified Harris Hip Score by 18.9 (Cohen’s d = 1.10), Non-arthritic Hip Score by 19.6 (Cohen’s d = 1.10), Hip Outcome Score-Sport by 25.6 (Cohen’s d = 1.15), and Visual Analogue Scale-Pain by −3.0 (Cohen’s d = −1.81). RTP averaged 81.4%, ranging from 73.3% to 90.0%. RTSP ranged from 40.6% to 81.0% with an overall OR of 83 (I²= 0.00). This meta-analysis found excellent patient-reported clinical outcomes at 1-year minimum follow-up after labral reconstruction in athletes, and a return-to-play rate of 81.4%. These data can help counsel athletes who undergo arthroscopic hip labral reconstruction.

## Introduction

Femoroacetabular impingement (FAI) commonly causes hip pain and labral damage in athletes [1, 2]. An intact labrum plays a critical role in hip stability and contact stress minimization [3]. For symptomatic, pre-arthritic FAI patients with labral tears, labral repair is the gold standard if there is enough intact and healthy labrum [4]. In cases where there is too little healthy labrum amenable for labral repair, labral reconstruction techniques have shown positive clinical outcomes [5, 6]. As athletes are a common group to suffer labral tears secondary to FAI, there is value in understanding how athletes recover and return to play (RTP) following labral reconstruction.

Current literature examining labral repair shows high rates of RTP, but results after labral reconstruction in athletes are not as well-established and summarized. The purpose of this study was to conduct a systematic review and meta-analysis on athletes undergoing arthroscopic hip labral reconstruction, focusing on postoperative (1) patient-reported outcomes (PROs) and (2) ability to RTP. This review will allow clinicians to offer their patients better insight into their recovery from labral reconstruction as they seek to RTP.

## Methods

The clinical question was developed using the PICO(T) model (Population, Intervention, Comparison or control, Outcome, and Time period) [7]. The question was ‘How have athletes (population) who underwent labral reconstruction (intervention), compared to their preoperative condition (comparison), reported outcomes and return to play changed (outcome) after follow-up greater than 1 year (time period).’ This review was conducted using the Preferred Reporting Items for Systematic Reviews and Meta-Analysis (PRISMA) guidelines [8]. The databases SCOPUS, Embase, OVID Medline, Cochrane Central Register of Clinical Trials, and clinicaltrials.gov were searched from their inception to 12/9/2023. Search terms included labral, reconstruction, femoroacetabular, and impingement within article title and abstract, and the results were correlated to the PICO(T) question.

Each article was identified for inclusion in this review by two intendent authors (D.C.I. and K.P.O). Inclusion criteria for this review required studies to report on (1) arthroscopic labral reconstruction in athletes using allografts or autografts, (2) PROs at baseline and at least 1-year postoperatively, and (3) RTP rate. RTP rate included an absolute rate of how often athletes returned to their sport as well as the RTP when considering factors unrelated to the surgical hip. Athletes were defined as patients who participated in any sport at the professional, collegiate, high school, recreational, or amateur level within 1 year of their first labral reconstruction surgery or planned to return to sport at the time of surgery. All studies underwent data extraction by two intendent authors (D.C.I. and K.P.O). Extracted data included demographics, concomitant procedures, PROs, RTP, and reason for stop of play. Across all included studies, labral reconstruction was performed when intraoperative assessment demonstrated that the native labrum was not amenable to repair: irreparable tears with complete disruption of longitudinal fibres, hypoplastic, or hypotrophic labra measuring ˂5 mm in width, or calcified labra. Both primary and revision labral reconstruction were included. Exclusion criteria for this review were studies that did not satisfy all 3 inclusion criteria, basic science studies, animal studies, case reports, cadaver studies, technique papers, guidelines, letters to the editor, abstracts, supplements, and non-English studies. In the case of repeated or overlapping cohorts, the more recent cohort, or study was used.

Additional quality assessment was performed for inclusion in this review. The Newcastle-Ottawa Scale (NOS) was used to assess the quality of non-randomized studies that were included in systematic reviews by awarding stars based on how well a non-randomized study fulfilled criteria proposed by the NOS [9]. A total of 9 stars were awarded based on selection (4 stars), comparability (2 stars), and outcomes (3 stars). Studies that earn ≥7 stars were considered ‘good’, 2–6 stars were ‘fair’, and <1 were ‘poor’ quality. This review included only studies that were awarded ‘good’ quality by the NOS.

### Data analysis

Statistics were performed using IBM SPSS (version 29). Continuous outcomes were combined and reported using means with standard deviations or ranges. Categorical variables were combined and reported using frequencies. Meta-analysis was performed, and forest plots were generated to report the results. For continuous data, Cohen’s d values were calculated to report the effect sizes of the studies. For binary data, log odds ratios (OR) were generated and then reported as OR. Tests for heterogeneity were performed and reported as I^2^. High I^2^ values indicate high levels of heterogeneity. *P*-values < .05 were considered statistically significant.

## Results

The PRISMA diagram is represented in Fig. 1. Database search yielded 430 records for review. A total of 213 duplicate studies were removed, then 217 study titles and abstracts were screened for inclusion. Around 57 studies had their full text evaluated. After evaluating full texts, four studies were included for data extraction and meta-analysis. The NOS scores for these four studies are reported in Supplemental Table 1.

**Figure 1:**
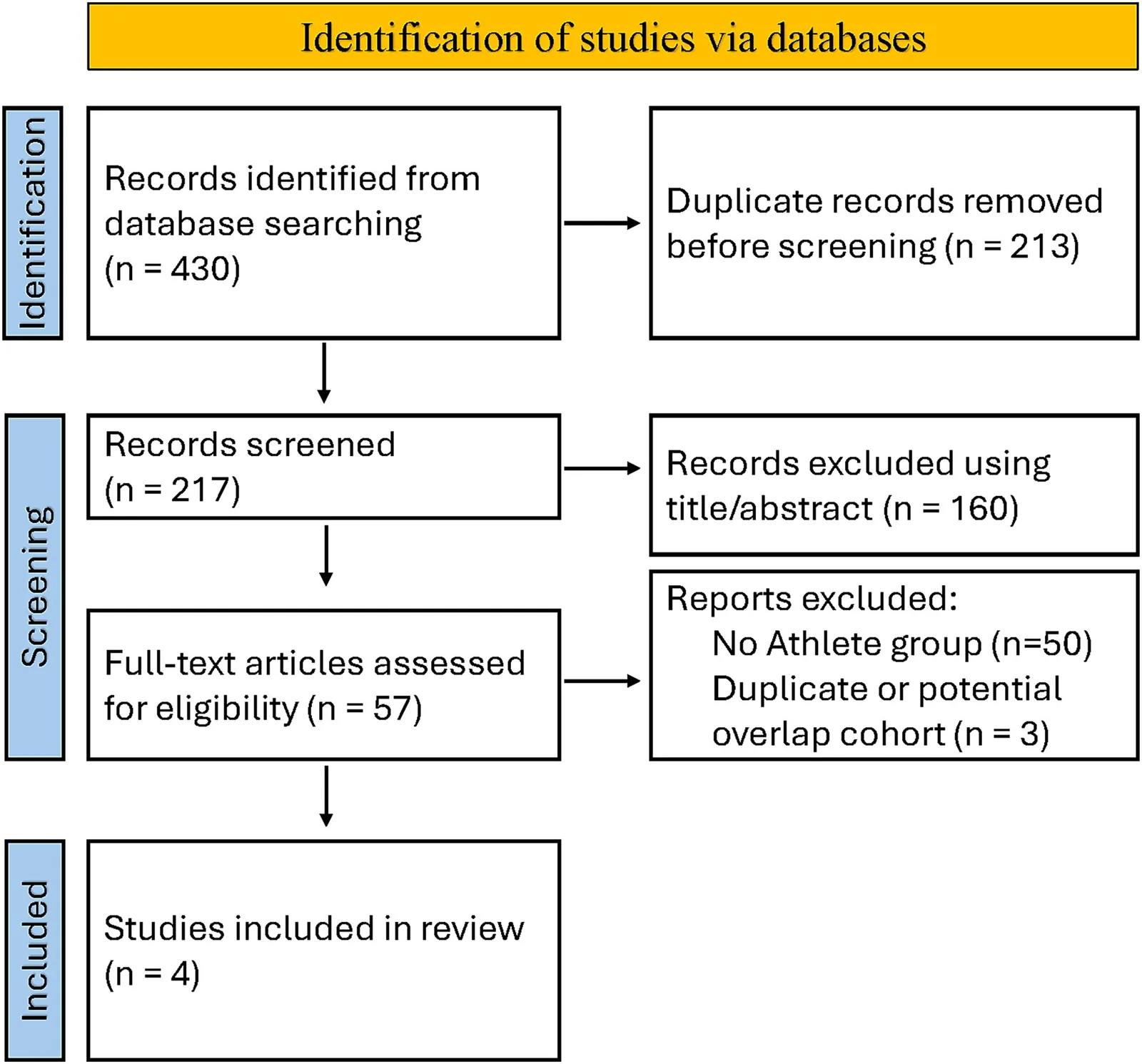
PRISMA diagram.

There were 126 hips across all included studies. The average age at labral reconstruction amongst all hips was 32.2 years and the average body mass index (BMI) was 25.6 kg/m^2^. Females made up 46.0% of all included hips and the average time to follow-up was 32.2 months. Both primary (61.9%) and revision (38.1%) reconstructions were included. Amongst the included studies, one used autografts exclusively, one used both autografts and allografts, and two used allografts exclusively. In total, 78.6% (n = 99) hips had labra reconstructed with allograft and 21.4% (n = 27) had labra reconstructed with autografts. Additional demographic data are listed in Table 1. Details on sport and level of play are listed in Table 4.

**Table 1:** Patient demographics.

Author	N hips (patients)	Percent primary	Percent revision	Mean age at labral reconstruction	Graft	Female %	Average BMI (Kg/m^2^)	Min follow-up (months)	Mean follow-up (months)
Boykin (2013) [10]	23 (21)	100	0	28.0 (19–41)	Iliotibial Band Autograft	0		12	41.4 (20–74)
Jimenez (2022) [11]	41 (41)	0	100	29.6 ± 9.7	Allograft & Autograft	65.9	25.4 ± 4.5	24	31.7 ± 9.3
Maldonado (2020) [12]	32 (32)	100	0	40.3 (15.5–58.7)	Ipsilateral Hamstring Tendon & Hamstring Allograft	43.8	27.9 (19.6–40.1)	12	26.4 (12.0–64.2)
Scanaliato (2020) [13]	30 (30)	76.67	23.33	30.4 (16–56)	Tensor Fascia Lata Allograft	56.6	23.4 (18.8–39.3)	24	24.3 (23.0–25.9)

The most common concomitant procedure was femoroplasty which was reported in all four studies and occurred in 90.0%–100% of labral reconstruction cases. Microfracture rate was reported in all four studies and occurred in 0%–39.1% of cases. Additional operative details are listed in Table 2.

**Table 2:** Concomitant procedures.

Author	N hips	Femoroplasty n (%)	Acetabuloplasty n (%)	Microfracture n (%)	Capsular repair n (%)	Ligamentum teres debridement n (%)	Iliopsoas fractional lengthening n (%)	Trochanteric bursectomy n (%)	Gluteus medius repair n (%)
Boykin (2013) [10]	23	21 (91.3)		9 (39.1)					
Jimenez (2022) [11]	30	29 (96.7)		3 (10.0)	21 (70.0)				
Maldonado (2020) [12]	32	32 (100)	32 (100)	3 (9.4)	17 (53.1)	7 (12.9)	8 (25.0)	6 (18.8)	4 (12.5)
Scanaliato (2020) [13]	30	27 (90.0)	15 (50.0)	0		2 (6.7)			

### Patient reported outcomes

The preoperative and final follow-up PROs included the modified Harris Hip Score (mHHS, n = 3), Hip Outcome Score- Sport Scale (HOS-Sport, n = 3), Visual Analogue Scale- Pain (VAS-Pain, n = 3), and the Non-arthritic Hip Score (NAHS, n = 2). All PROs reported in each study demonstrated statistically significant improvement (*P* < .05) after labral reconstruction (Table 3). Pooled weighted averages and forest plots for PROs showed improved outcomes and large Cohen’s d values (Fig. 2). The mHHS improved by an average of 18.9 (range, 16.4–21.9) with a postoperative value of 81.2 (range, 76.2–86.2) and Cohen’s d = 1.18 which was above the minimal clinically important difference (MCID) of 8 and patient acceptable symptomatic state (PASS) of 74. The HOS-Sport improved by an average of 25.6 (range, 20.8–28.3) with a postoperative value of 68.3 (range, 56.4–78.9), and Cohen’s d = 1.15 which was above the MCID of 6 but below the range of PASS of (72.1–75). The VAS-Pain improved by an average of −3.0 (range, −4.5 to −2.3) with a postoperative value of 2.3 (range, 1.2–3.5) and Cohen’s d = −1.81 which is above the MCID of −1.4. The NAHS improved by an average of 19.6 (range, 11.1–16.7) with a postoperative value of 80.5 (range, 74.8–87.7) and Cohen’s d = 1.18 which was above the MCID of 8.7 but below the range of PASS (81.9–85.6). Pooled weighted average improvements are shown in Fig. 3.

**Table 3:** Patient reported outcomes.

Author	Subgroup	Hips	Mean follow-up months	mHHS	HOS-sport	NAHS	VAS-pain
Boykin (2013) [10]	Preoperative Postoperative Mean difference (95% CI) *P* value	23 17	41.4 (20–74)	67 84 16.4 (2–30) **0.026**	56 77 20.8 (6–35) **0.009**		
Jimenez (2022) [11]	Preoperative Postoperative Mean difference (range) *P* value	41 41	31.7 ± 9.3	58.2 ± 12.3 (31–81) 76.2 ± 18.4 (26–100) 17.4 ± 20.8 (−15–69) **< .001**	31.3 ± 17.3 (0–72) 56.4 ± 25.7 (8.3–100) 24.3 ± 27.4 (−33.3–100) **< .001**	56.3 ± 13.5 (25–81) 74.8 ± 18.4 (27.5–100) 16.7 ± 18.1 (−21.3–61.3) **< .001**	5.8 ± 2 (0–9.2) 3.5 ± 2.4 (0–9) −2.3 ± 2.8 (−5.9–8.1) **< .001**
Maldonado (2020) [12]	Preoperative Postoperative Mean difference (95% CI) *P*-value	32 32	26.4 (12.0–64.2)	64.4 ± 15.1 86.2 ± 17.1 21.9 (−14–50) **< 0.001**	47.6 ± 25.6 78.9 ± 30.2 28.3 (−30.6–77.8) **< 0.001**	66.6 ± 16.9 87.7 ± 17.6 11.1 (−11.3–52.5) **< 0.001**	4.4 ± 1.8 1.8 ± 2.1 −2.6 (−8–2.6) **< 0.001**
Scanaliato (2020) [13]	Preoperative Postoperative Mean difference (95% CI) *P*-value	30 30	24.3 (23.0–25.9)				5.7 ± 1.6 1.2 ± 1.2 −4.5 (−5.2–3.8) **< 0.001**

Bold values indicate statistically significant *P* value < 0.05.

**Figure 2:**
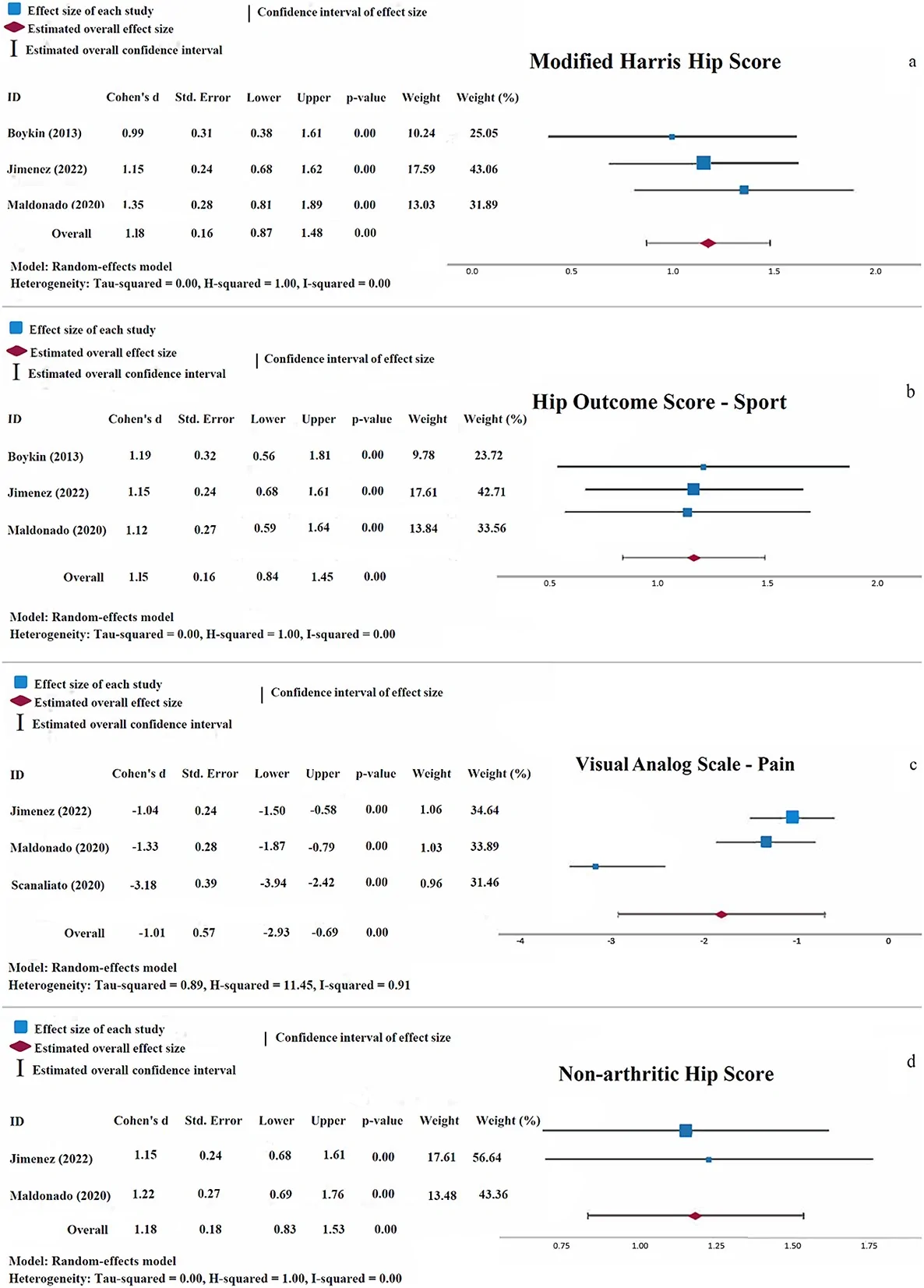
(a)–(d) Patient reported outcomes Forest plots.

**Figure 3:**
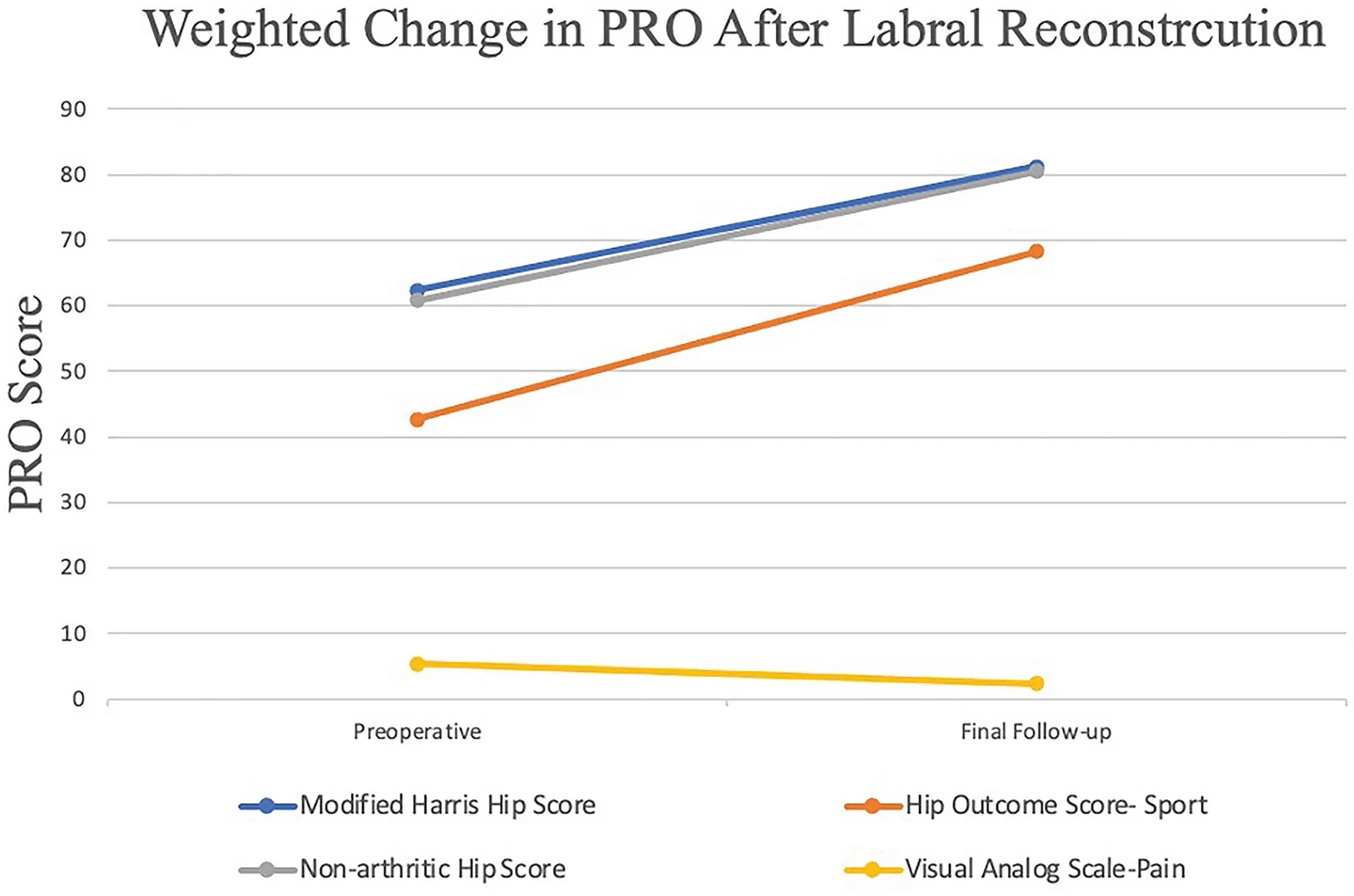
Weighted change in patient reported outcomes after labral reconstruction.

### Return to play

RTP rates are listed in Table 4. The pooled weighted average of RTP across the four studies was 73.5%. When including those patients who stopped sport due to concerns independent of their surgical hip, this average rate increased to 81.4% (range, 73.3%–90.0%, Table 4). Across all studies, 20.4% of athletes competed professionally whilst the remaining 79.6% competed at the amateur level, including college, high school, and recreational sports. A forest plot of all four studies revealed that the odds ratio for RTP compared to the preoperative condition was 247 and had an I^2^ value of 0.00, indicating very low heterogeneity (Fig. 4a). Three studies looked at level of play and reported that between 40.6% and 81.0% of athletes achieved the same or higher level of return to same level of play (RTSP) compared to the preoperative condition with an odds ratio of 83 and an I^2^ value of 0.00 (Fig. 4b). The highest rate of RTSP was reported in a cohort consisting of exclusively professional athletes (81.0%) whilst the lowest RTSP was reported in a cohort of college, high school, and recreational athletes (40.6%).

**Table 4:** Return to play.

Author	Athletes n	Return to play (%)	Stopped play due to non-ipsilateral hip factors (%)	Returned to play or stopped play due to non-ipsilateral hip factors (%)	Returned to same or higher level of performance (%)	Level of competition (n)	Sport (n)
Boykin (2013) [10]	21	85.7	0.0%	85.7%	81.0%	Professional (21)	Soccer (7), Hockey (5), Football (4), Ski (2), Baseball (1), Basketball (1), Ice Skate (1)
Jimenez (2022) [11]	30	46.7%	26.7%	73.3%		Professional (1), College (4), High School (6), Amateur (2), Recreational (17)	Run (7), Dance (3), Hockey (3), Cycle (2), Basketball (1), Football (1), Soccer (1), Swim (1), Volleyball (1), Other (10)
Maldonado (2020) [12]	32	78.1%	0.0%	78.1%	40.6%	College, High School, Recreational, Amateur	Run (11), Cycle (4), Dance (3), Golf (3), Baseball (2), Martial Art (2), Soccer (1), Hockey (1), Ski (1), Swim (1), Tennis (1), Equestrian (1), Weightlift (1)
Scanaliato (2020) [13]	30	86.7%	3.3%	90.0%	57.70%	Professional (1), College (7), High School (17), Amateur or Recreational (5)	Run (11), Lacrosse (3), Tennis (3), Dance (3), Golf (2), Tactical Athlete (2), Soccer (2), Wakeboard (2)

**Figure 4:**
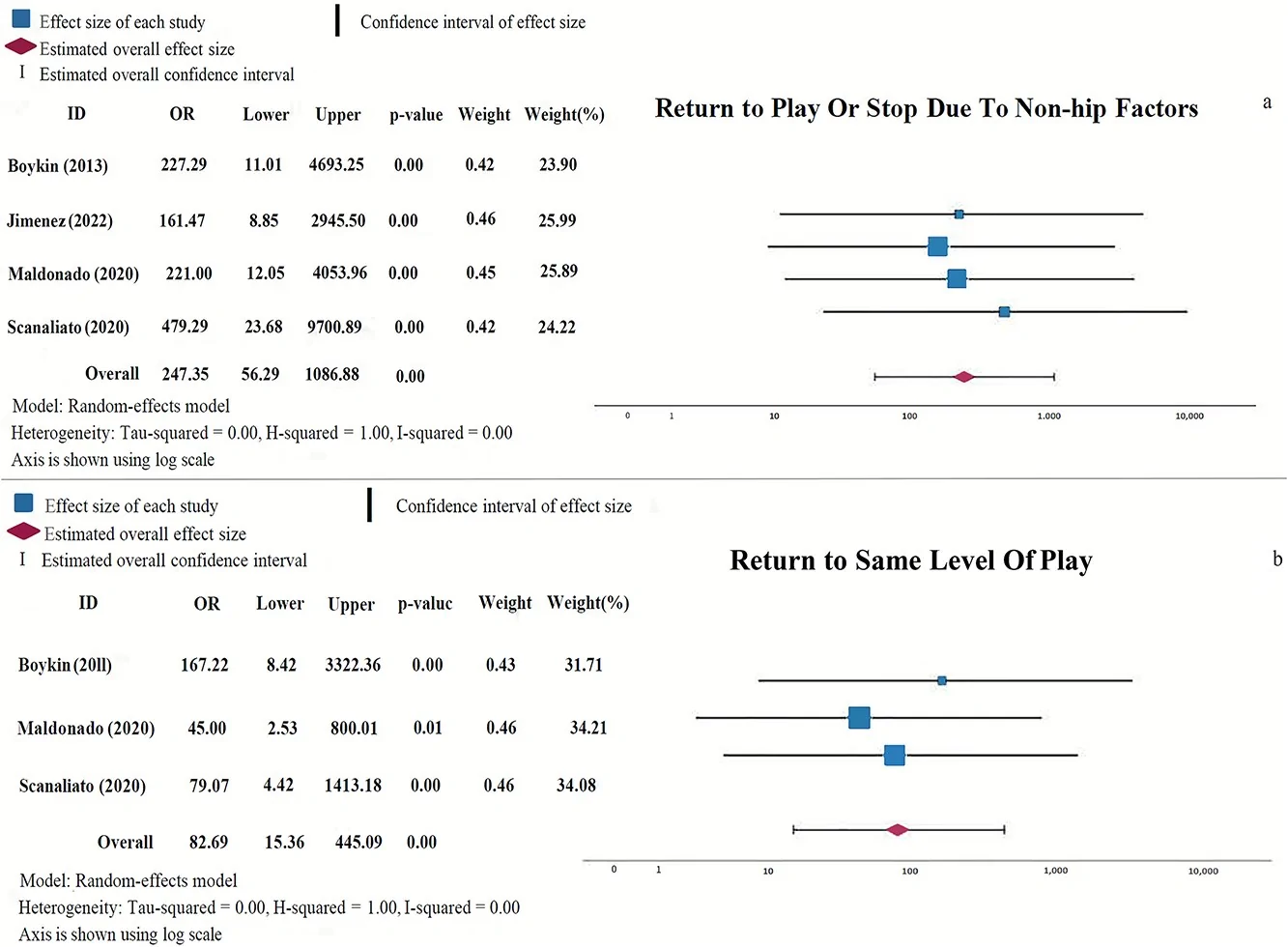
(a) and (b) RTP Forest plots.

## Discussion

This review examined the current literature on patient reported outcomes and RTP in athletes following labral reconstruction. Our analysis showed that athletes undergoing labral reconstruction across multiple levels of competition and a variety of sports demonstrated improved patient reported outcomes and positive RTP rates. These findings are important because previous systematic reviews have analyzed outcomes in athletes who receive hip arthroscopy with labral repair, but not in athletes with labral pathology significant enough to warrant labral reconstruction [14, 15].

Each of the included studies reported PROs and all studies found significant improvement in all PROs compared to their preoperative values. Additionally, the mHHS, NAHS, HOS-Sport, and VAS-Pain all improved beyond the MCID in each study. The postoperative weighted pooled average for mHHS (81.2) was above the PASS (74) whilst the NAHS (80.5) and HOS-Sport (68.3) were below the range of PASS (81.9–85.6 and 72.1–75, respectively) [16–18]. This demonstrates that athletes report clinical improvement at a minimum of 1 year after labral reconstruction. The improvements seen in these athletes PROs are in accordance with the current literature, including a systematic review of labral reconstruction outcomes in a standard patient population that similarly showed PASS achieved in the pooled weighted average mHHS [19]. Furthermore, similar HOS-Sport results have been reported in labral repairs, where athletes demonstrated higher mean HOS-Sport compared to non-athletes yet showed a slightly lower and statistically non-significant rate of achieving substantial clinical benefit on the postoperative HOS-Sport score [20]. Another systematic review in a standard patient population demonstrated that labral reconstruction led to significant postoperative improvements in PROs, with outcomes statistically equivalent to those achieved with labral repair [21]. This further supports the study’s findings that athletes with significant labral pathology requiring reconstruction, rather than repair, can achieve postoperative outcomes that are favourable and similar to labral repair. Amongst the cohorts in the included studies, labral reconstruction was performed using autografts in one article, allografts and autografts in another article, and allografts exclusively in two articles. A recent systematic review found no significant differences in most PRO by graft source in labral reconstruction; thus, graft choice is unlikely to substantially impact the PRO findings in this review [22].

The average rate of RTP or stop of play due to non-surgical-hip factors was 81.4% (73.3–90.0%) with one study exclusively reporting on professional athletes whilst the remaining three studies reported on athletes ranging from recreational to professional. A separate systematic review of RTP in recreational athletes and elite athletes after hip arthroscopy for FAI, labral tears, or chondral injuries reported a similar average RTP rate of 84.6% [23]. Elite athletes report higher rates of RTP after hip arthroscopy for FAI, labral tears, or chondral injuries with one systematic review reporting average RTP rate of 93.9% [15]. Furthermore, this systematic review’s finding that return to sport (RTS) after labral reconstruction is comparable to RTS after labral repair is supported by a propensity-matched analysis demonstrating RTS rates of 82.4% for labral reconstruction and 82.8% for labral repair [24].

Three studies included in this review reported on the rate that athletes return to previous level of performance or greater level of performance after labral reconstruction. The highest rate of RTSP (81.0%) was reported in a cohort of exclusively professional athletes whilst studies with a range of athletes reported rates of 57.7% and 40.6%. Similar findings have been reported in other studies that show professional athletes are more likely to return to previous level of performance compared to recreational athletes despite competing at a higher level [25]. The literature shows that RTSP in non-professional athletes is substantially lower than in professional athletes, with an RTSP rate of 57.1% reported after labral repair which is consistent with the findings of this systematic review amongst cohorts composed primarily of non-professional athletes [26].

There were several limitations to this systematic review. The number of data points across the included studies in this meta-analysis allowed for forest plots but not funnel plots. Examination of the four included studies’ cohorts showed a great deal of variation in the level of athlete competition both within and across the included studies. Furthermore, there was also a great deal of variation in the sports played by athletes in each study which can affect generalizability of this review’s findings. The demands placed on a runner’s hip, a golfer’s hip, and a martial artist’s hip vary greatly, yet are reported in the included studies under the umbrella term of athlete. The PROs of HOS-Sport, mHHS, and VAS-Pain were reported in three of the four included studies, with NAHS reported in two of the four studies. As more studies emerge, we look forward to additional opportunities to more effectively pool PROs in future meta-analyses. There was heterogeneity in the graft used for the different cohorts’ labral reconstruction. However, other systematic reviews have demonstrated no significant differences in outcomes based on graft type in labral reconstruction [19]. The incorporated studies included athletes who underwent labral reconstruction as both primary and revision procedures but presented data in a manner that prevented a meaningful subgroup analysis comparing these groups. To provide a comprehensive overview of the current literature on labral reconstruction outcomes in athletes, both primary and revision cases were analyzed together, consistent with the approach of one of the included studies. The limited available literature also precludes other subgroup analyses. Nonetheless, the findings of this systematic review suggest that labral reconstruction is an effective treatment option in athletes. As labral reconstruction in this population becomes more common and future studies emerge, we are eager for more data that will allow for detailed subgroup analyses and further refinement of outcomes. The remaining limitations are those inherent in all systematic reviews: selection bias, inadequate blinding, attrition bias, selective outcome reporting, and publication bias. A larger number of articles on labral reconstruction in athletes would have been beneficial in creating funnel plots and minimizing the inherit bias of systematic reviews.

## Conclusion

This meta-analysis and systematic review showed that despite labral reconstruction occurring in patients with severe labral injury, athletes report excellent outcomes and similar rates of RTP compared to arthroscopic hip labral repair. An average of 81.4% of athletes returned to play or stopped play due to factors unrelated to their surgical hip at 1-year minimum follow-up. RTSP in cohorts of only professional athletes was 81.0% whilst RTSP in cohorts of primarily non-professional athletes ranged from 40.6%–57.7%. Each included study in this review demonstrated statistically and clinically significant improvement in mHHS, HOS-Sport, NAHS, and VAS-pain at follow-up. Labral reconstruction in athletes is an effective way to treat severe labral injury, demonstrating favourable outcomes and RTP at 1-year minimum follow-up.

## Supplementary Material

Supplemental_Table_1_hnaf067
