# Supplementary material for: Patient reported outcomes and return to play after arthroscopic hip labral reconstruction in athletes: a systematic review and meta-analysis
Source: J Hip Preserv Surg. 2025 Nov 24;13(3):259–67. doi: 10.1093/jhps/hnaf067 (PMC13653053; doi:10.1093/jhps/hnaf067)
Supplement: Supplementary file 1 [file supplemental_table_1_hnaf067.docx]

| **Supplemental Table 1** | | | | | | | | | |
| --- | --- | --- | --- | --- | --- | --- | --- | --- | --- |
| **Newcastle-Ottowa Scale** | | | | | | | | | |
| Author | Q1 | Q2 | Q3 | Q4 | Q5 | Q6 | Q7 | Q8 | Total* |
| Boykin (2013)^10^ | * |  | * | * | * | * | * | * | 7 |
| Jimenez (2022)^11^ | * | * | * | * | ** | * | * | * | 9 |
| Maldonado (2020)^12^ | * |  | * | * | * | * | * | * | 7 |
| Scanaliato (2020)^13^ | * |  | * | * | * | * | * | * | 7 |
